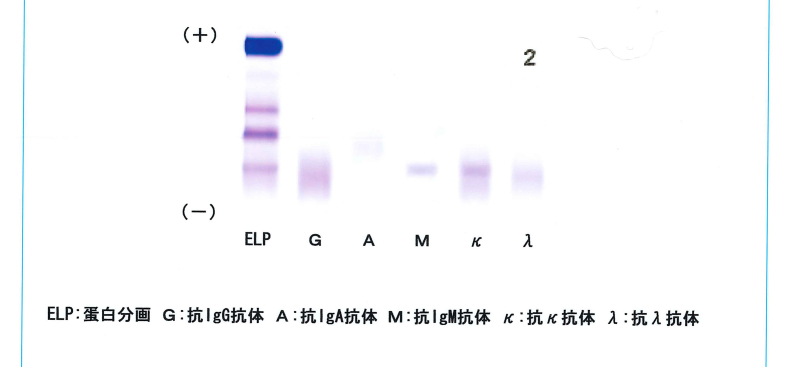# Anti-myelin-associated-glycoprotein neuropathy successfully treated with tirabrutinib

**DOI:** 10.1016/j.heliyon.2022.e10928

**Published:** 2022-10-05

**Authors:** Hajime Yasuda, Yuji Tomizawa, Sakiko Harada, Makoto Sasaki, Norio Komatsu, Jun Ando, Nobutaka Hattori, Miki Ando

**Affiliations:** a Department of Hematology, Juntendo University School of Medicine, Tokyo, Japan; b Department of Neurology, Juntendo University School of Medicine, Tokyo, Japan; c Laboratory for the Development of Therapies Against MPN, Juntendo University School of Medicine, Japan; d Department of Advanced Hematology, Juntendo University School of Medicine, Japan; e Department of Cell Therapy and Transfusion Medicine, Juntendo University School of Medicine, Japan

**Keywords:** Anti-MAG antibody, Ibrutinib, BTK inhibitor, Lymphoplasmacytic lymphoma, IgM MGUS, Rituximab, Bruton tyrosine kinase inhibitor, Plasmapheresis, SGPG glycolipid, Sensory-motor type polyneuropathy

## Abstract

**Background:**

Anti-myelin-associated-glycoprotein (MAG) neuropathy is a distal, predominantly demyelinating, sensory or sensory-motor polyneuropathy most often developing in the context of an IgM-type monoclonal gammopathy due to monoclonal gammopathy of undetermined significance or lymphoplasmacytic lymphoma. Rituximab is considered standard therapy for treatment naïve patients, but optimal treatment methods for relapsed/refractory patients have not been established.

**Case presentation:**

We demonstrate that tirabrutinib, a second-generation Burton kinase inhibitor, led to drastic improvements of polyneuropathy that were affirmed by nerve conduction studies in a rituximab-refractory anti-MAG neuropathy patient. Tirabrutinib continues to give excellent disease control with no apparent adverse events at 11 months since initiation, and the patient remains free of plasmapheresis sessions which were originally mandatory.

**Conclusion:**

Tirabrutinib is an extremely promising treatment option for anti-MAG neuropathy.

## 1 Introduction

Anti-myelin-associated-glycoprotein (MAG) neuropathy is a distal, predominantly demyelinating, sensory or sensory-motor polyneuropathy most often developing in the context of an IgM-type monoclonal gammopathy due to monoclonal gammopathy of undetermined significance (MGUS) or lymphoplasmacytic lymphoma (LPL). Treatment is targeted towards the anti-MAG antibody producing B-cell clone and rituximab monotherapy is most often used as first-line therapy. However, there is no standard treatment approach for patients failing rituximab, and extrapolation of treatment methods for LPL have often been incorporated [1]. We report a rituximab-refractory anti-MAG neuropathy patient successfully treated with tirabrutinib, a second generation Bruton tyrosine kinase (BTK) inhibitor.

## 2 Case presentation

**Figure 1:**
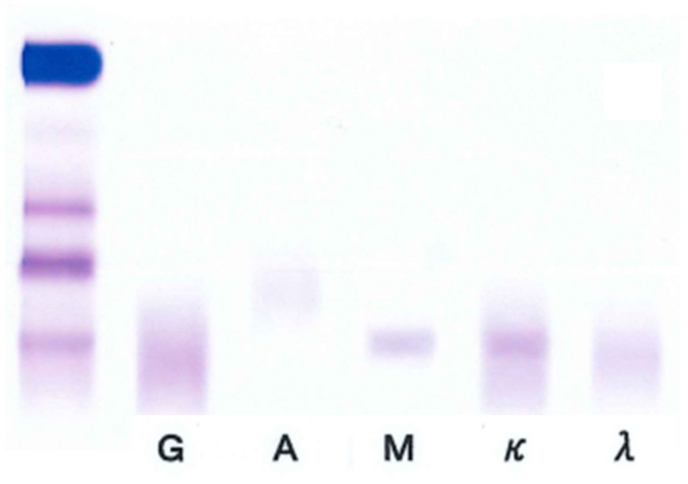
Immunofixation results. Abbreviations: G: anti-IgG antibody; A: anti-IgA antibody; M: anti-IgM antibody; κ: anti-kappa antibody; λ: anti-lambda antibody. Original unedited image results are available as a supplementary file.

**Figure 2:**
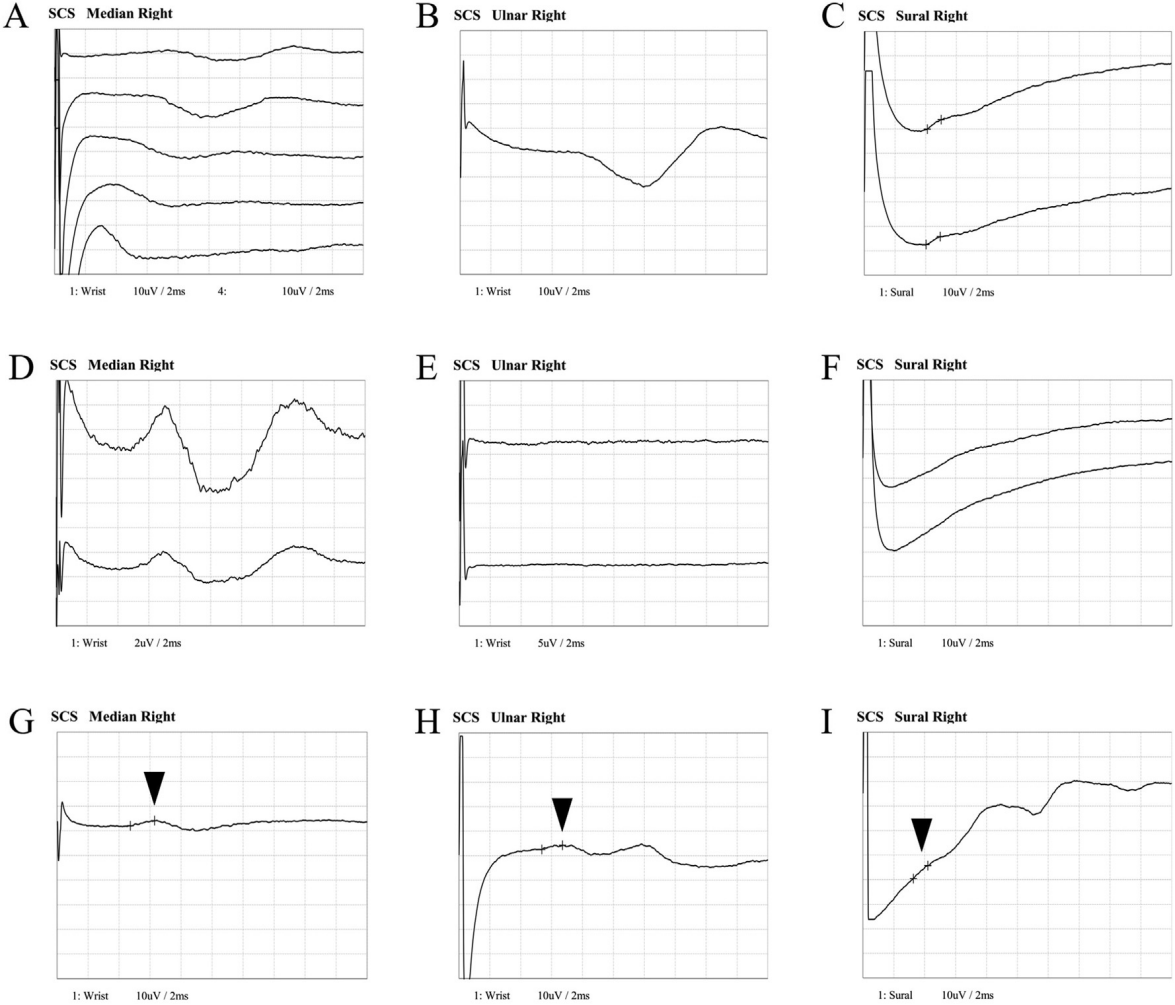
Nerve conduction studies at various time points. Sensory nerve action potentials (SNAPs) of median, ulnar, and sural nerves. A, B, C. Nerve conduction studies at diagnosis showed loss of SNAPs on median and ulnar nerves, and small SNAPs could be observed on the sural nerve. D, E, F. Six months from rituximab treatment and before tirabrutinib administration. SNAPs were absent on median, ulnar, and sural nerves. G, H, I. Seven months after initiation of tirabrutinib. Small SNAPs could be confirmed on the median, ulnar, and sural nerves (arrow heads).

A 57-year-old man was referred to our hospital in July 2020 due to numbness and tingling of both feet and ataxic gait. Distal limb muscle weakness and both superficial and deep sensory impairment of the hands and feet were evident. Immunofixation tests revealed an IgM-kappa type monoclonal protein (Figure 1), and anti-MAG and anti-sulfated glucuronyl paragloboside (SGPG) antibodies both showed high titers of 1:204,800 (reference range: <1:1600) and 1:819,200 (reference range: <1:3200), respectively. Anti-MAG and anti-SGPG antibodies were detected by enzyme linked immunosorbent assay (ELISA), and positive results were confirmed by MAG Western blot methodology by MAG ‘Dual Antigen’™ Autoantibody Test, Athena Diagnostics. Serum IgM elevation was mild at 303 mg/dL (reference range: 33–190 mg/dL) and protein electrophoresis showed a very small M-spike, implicating a small monoclonal protein quantity. Nerve conduction studies showed disappearance of sensory nerve action potentials (SNAPs) on the right median and right ulnar nerves, and reduced amplitude on the right sural nerve (3.90 μV) (Figure 2, A-I). Bone marrow evaluation was unremarkable with no increase of plasma/lymphoplasmacytic cells and no kappa/lambda light chain deviation upon flow cytometry, and thus IgM MGUS with anti-MAG neuropathy was diagnosed. Due to rapidly worsening polyneuropathy, emergency plasmapheresis was initiated in September 2020 and continued for 5 sessions every 3–4 days. Polyneuropathy improved, and he became able to walk on his own if aided by a cane. Treatment with rituximab was planned, but in advance, polyneuropathy rapidly exacerbated to the extent to which he could not walk by himself, and 7 sessions of plasmapheresis were carried out from the end of November 2020, again leading to improvement of polyneuropathy. Subsequently, 4 weekly administrations of rituximab at 375 mg/m^2^ were carried out from the end of December 2020. However, polyneuropathy exacerbation was again observed, and periodic plasmapheresis had to be resumed approximately a week following the fourth administration of rituximab. Thereafter, exacerbations and improvements of polyneuropathy before and after plasmapheresis sessions were repeatedly observed for approximately 6 months, and thus rituximab therapy was judged to have failed. Tirabrutinib was chosen as second-line treatment, and oral administration at 480mg/day was initiated from early July 2021. The last session of plasmapheresis was carried out in late July 2021, but for the first time, exacerbations of polyneuropathy did not recur thereafter. As of May 2022, tirabrutinib is continued in the patient with no apparent adverse events at 11 months, and the patient's neuropathy has improved to the point to which he can walk unaided and uses a cane just for reassurance. Nerve conduction studies also showed improvement of SNAPs (right median nerve: 2.10 μV, right ulnar nerve: 1.70 μV, right sural nerve: 5.30 μV) (Figure 2). Although immunofixation tests still demonstrate the presence of an IgM-kappa type monoclonal protein, serum protein electrophoresis shows improvement with a barely detectable M-spike. Anti-MAG and anti-SGPG antibody titers have also both declined to 1:102,400 and 1:409,600, respectively.

## 3 Discussion

Rituximab monotherapy is considered standard of care for treatment-naïve anti-MAG neuropathy patients, but treatment for rituximab-refractory patients remains to be established. Furthermore, only 30–50% of patients with anti-MAG neuropathy respond to rituximab, and thus a significant number of patients are forced to directly proceed to second-line therapy [2]. Because the underlying monoclonal gammopathy responsible for generating the anti-MAG antibodies is typically IgM MGUS or LPL, second-line treatment is usually chosen by extrapolating treatment methods for LPL. Tirabrutinib is an oral second-generation BTK inhibitor with higher BTK selectivity and fewer off-target effects compared to ibrutinib [3]. Tirabrutinib was approved in Japan in August 2020 for the treatment of WM and LPL, subsequent to approval for treatment of relapsed/refractory primary central nervous system lymphoma (PCNSL) [4]. A Japanese phase 2 trial administering 480mg/day of tirabrutinib to 27 Waldenström macroglobulinemia (WM) patients reported a major response rate of 93%, 2-year PFS rate of 92.6%, and 2-year OS of 100% [3]. Based upon these results, we chose tirabrutinib monotherapy as second-line treatment for the presented patient. Although efficacy of ibrutinib has been demonstrated in 3 anti-MAG neuropathy patients in a previous report, this is the first to report efficacy of tirabrutinib in a rituximab-refractory anti-MAG neuropathy patient [5]. Other than WM, tirabrutinib has also been reported to be effective for treatment of PCNSL, mantle cell lymphoma, and chronic lymphocytic leukemia, and thus application of its use can be expected to expand in the future for a broad range of B-cell malignancies [6, 7, 8]. Most frequent common adverse events of BTKs include bleeding, skin rash, and various cytopenias, but no adverse events were seen in the presented patient [3, 9, 10]

MAG is a transmembrane glycoprotein located between Schwan cells and axons, and MAG-deficient mice have shown degeneration of both myelin and axons. Similar findings and axonal protective effects of MAG have been reported in humans [2]. Diagnosis of anti-MAG neuropathy basically depends on demonstration of anti-MAG antibodies in patient serum. Because anti-MAG antibodies also recognize SGPG glycolipid, anti-SGPG assays have been used as a surrogate for diagnosing anti-MAG neuropathy. However, anti-MAG antibodies have much higher affinity towards MAG compared to that towards SGPG, and therefore anti-MAG assays are preferable [2]. In the presented case, both anti-MAG and anti-SGPG assays were carried out, and improvements were seen in both assays after tirabrutinib treatment. Although it has been reported that there is no direct association between anti-MAG antibody levels and severity of neuropathy at disease diagnosis, a retrospective study including 410 patients with anti-MAG neuropathy demonstrated that a relative decrease in serum anti-MAG antibodies subsequent to therapy is strongly associated with clinical response, and a reduction of at least 50% of anti-MAG antibody titers compared with pretreatment titers was a useful indicator of response. Our patient also presented data in parallel with 50% reductions of both anti-MAG and anti-SGPG antibody titers along with clear improvements of polyneuropathy [2, 11].

One limitation of this report is that tirabrutinib was administered subsequent to rituximab, and it could be questioned that rituximab may have also contributed to response. However, we waited 6 months with no signs of improvement after rituximab therapy before administrating tirabrutinib, and thus response was thought to be mostly if not entirely attributed to tirabrutinib. Another limitation is that although unavoidable due to rapid exacerbation of neuropathy, plasmapheresis was resumed approximately a week after the fourth administration of rituximab, and a significant amount of rituximab may have been eliminated through plasmapheresis. Therefore, it can be argued that rituximab may have been effective if not for early resumption of plasmapheresis. However, robust B-cell depletion has been reported in patients treated with plasmapheresis shortly after rituximab administration, and thus the effects of plasmapheresis on outcome are probably minimal [12]. Plasmapheresis has been reported to be effective in cases with rapid neurologic exacerbations by efficiently removing serum anti-MAG antibodies, as was observed in the presented case, but of course this brings about only temporary remission, and treatment should basically be targeted at the B-cell clone responsible for producing the anti-MAG antibodies [13].

## 4 Conclusion

In conclusion, tirabrutinib administration continues to provide excellent disease control at 11 months after initiation in a rituximab-refractory anti-MAG neuropathy patient, and no adverse events have been observed. Treatment methods for anti-MAG neuropathy have not been well established, and BTK inhibitors including tirabrutinib are a promising treatment approach.

## Statement of ethics

Written informed consent was obtained from the patient for publication of this case report. This study was approved by the ethics committee of Juntendo University School of Medicine (IRB#17-072).

## Declarations

### Author contribution statement

All authors listed have significantly contributed to the investigation, development and writing of this article.

### Funding statement

This research did not receive any specific grant from funding agencies in the public, commercial, or not-for-profit sectors.

### Data availability statement

Data will be made available on request.

### Declaration of interests statement

The authors declare no conflict of interest.

### Additional information

No additional information is available for this paper.

## Figures and Tables

**supplementary file:**